# Warming, Snow Exclusion, and Soil Type Alter the Timing of Plant and Soil Activity and Associated Nutrient Losses

**DOI:** 10.1111/gcb.70447

**Published:** 2025-08-19

**Authors:** Stephanie M. Juice, Paul G. Schaberg, Alexandra M. Kosiba, Carl E. Waite, Gary J. Hawley, Deane Wang, Julia N. Perdrial, E. Carol Adair

**Affiliations:** ^1^ Department of Biology West Virginia University Morgantown West Virginia USA; ^2^ Rubenstein School of Environment and Natural Resources, University of Vermont Burlington Vermont USA; ^3^ USDA Forest Service Northern Research Station Burlington Vermont USA; ^4^ University of Vermont Extension South Burlington Vermont USA; ^5^ Department of Geography and Geosciences University of Vermont Burlington Vermont USA; ^6^ Gund Institute for Environment, University of Vermont Burlington Vermont USA

**Keywords:** climate change, infrared warming, mesocosm, plant‐microbe asynchrony, soil freezing, soil nutrient loss, soil texture

## Abstract

In seasonally snow‐covered ecosystems, changing temperatures and snowpack dynamics under climate change have increased the occurrence and duration of soil temperatures that support microbial activity during plant dormancy. During these periods of microbial activity without plant activity (i.e., plant‐microbe asynchronies), soil nutrients that build up are vulnerable to leaching loss, with potentially important consequences for ecosystem productivity. Furthermore, asynchronies likely do not occur uniformly in space; rather, their occurrence may be modulated by subsurface characteristics. Soil texture, for example, moderates biogeochemical cycles and water holding capacity, and could mitigate or exacerbate nutrient losses during plant‐microbe asynchronies. Here, we quantified how climate change treatments and soil characteristics alter the synchrony of plant and microbial activity, and the associated impacts on leaching of soil nutrients—carbon, nitrogen, phosphorus—and cations prone to mobilization following environmental perturbation—calcium, magnesium, and aluminum. To do this, we conducted a forest sapling mesocosm experiment that imposed replicated warming and snow exclusion treatments on two soils. To estimate the extent and effect of asynchrony, we measured soil temperature and plant phenology over 2 years to develop an index for asynchrony duration, which we correlated with measured nutrient and cation leachate losses. We found that warming consistently increased the duration of plant‐microbe asynchrony, with an average increase of 25% across the experiment. Snow exclusion shortened asynchrony duration by 8% on coarse soils in the second year of the experiment. Climate treatments generally elevated nutrient losses from fine but not coarse soils during asynchronies. Longer asynchronies resulted in increased carbon, nitrogen, and magnesium losses, with variation across time, soil type, and nutrient. Our results demonstrate that longer periods of microbial activity in the absence of plant uptake generally compound nutrient losses, but the magnitude of these losses depends on soil type and individual nutrients.

## Graphical abstract

As winters warm, periods with warm soils that support rapid microbial activity while plants are dormant are increasingly frequent in ecosystems with seasonal snow. Soil nutrients, which are critical for plant growth, are vulnerable to leaching loss during these plant‐microbe asynchronies. These asynchronies have unknown impacts on nutrient leaching that likely vary across soil types. We conducted an experiment using large mesocosms with two soils and three climate treatments (control, warming, snow‐exclusion) to address this knowledge gap. We found that asynchronies increased with warming, decreased with snow exclusion, and that their length was correlated with carbon and nitrogen losses.

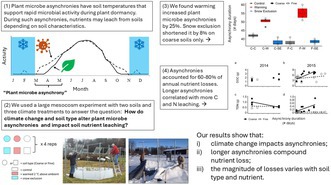

## 1 Introduction

In northern climates, the occurrence of soil temperatures that support rapid microbial activity during plant dormancy is increasing (Campbell et al. 2005; Contosta et al. 2017) due to the earlier arrival of warm temperatures in spring, delayed onset of cold temperatures in fall, and more frequent mid‐winter thaws (Demaria et al. 2016; Hayhoe et al. 2007; Henry 2008). In forests, rapid soil warming occurs in concert with snowmelt (Groffman et al. 2012; Molotch et al. 2009), creating warm, moist conditions that can accelerate microbial activity (Curiel Yuste et al. 2007). While plants are responding to warming temperatures (Polgar and Primack 2011), with spring leaf out occurring earlier (Keenan et al. 2014; Polgar et al. 2014) and fall senescence occurring later (Jeong et al. 2011), their phenology is controlled by a suite of environmental cues including photoperiod, timing of precipitation, and accumulation of growing degree days (Inouye 2022). As such, plant phenological shifts are controlled by some factors that vary with climate change (e.g., temperature, precipitation) and some that are invariant (e.g., photoperiod; Inouye 2022).

Conversely, soil microbial activity is controlled by temperature along with water and substrate availability (Curiel Yuste et al. 2007), conditions which are readily met during periods of thaw and lengthened shoulder seasons. Because soil microbes can respond more immediately to soil temperatures than plants, the frequency and duration of periods with soil microbial activity during plant dormancy (herein, plant‐microbe asynchronies *sensu* vernal asynchrony in Groffman et al. 2012) may increase under climate change (Contosta et al. 2017; Groffman et al. 2012). During plant‐microbe asynchronies, microbial activity leads to the buildup of soil nutrients that, in the absence of plant uptake, are vulnerable to leaching loss (Brooks et al. 1998; Muller and Bormann 1976; Tierney et al. 2001). While a previous study found large increases in carbon (C) loss to the atmosphere from a forest during an abnormally warm winter to spring transition (Sanders‐DeMott et al. 2020), it remains uncertain if protracted plant‐microbe asynchronies due to climate change will increase ecosystem losses of C and other nutrients via soil leaching (Contosta et al. 2017; Groffman et al. 2012).

Ecosystem properties have the capacity to mitigate or exacerbate the impacts of climatic changes on nutrient cycling and losses. For example, soil texture strongly influences nutrient losses by affecting the ability of soils to retain water and nutrients (Cosby et al. 1984; Jawson and Niemann 2007; Jenny 1980; Silver et al. 2000). Soil moisture can modify the effects of warming on soil temperature (Subin et al. 2013) and regulate microbial activity (Prado and Airoldi 1999; Tiwari et al. 1987), including decomposition, soil respiration, nitrogen (N) mineralization, and denitrification (Hamarashid et al. 2010; Silver et al. 2000; Xu et al. 2016). Many of the basic chemical attributes of soils that shape biological activity also vary across soil texture, including N and phosphorus (P) availability, soil organic matter pools (Hamarashid et al. 2010; Silver et al. 2000), and nutrient leaching rates (Tahir and Marschner 2017). In winter, soil texture affects the extent and severity of soil freezing (Fuss et al. 2016) with impacts for aggregate stability (Lehrsch et al. 1991) and water availability (and thus microbial activity; Gray et al. 1985; Ritchie 1981). Furthermore, variation in soil characteristics among watersheds and forest stands has been found to alter snow melt chemistry (Fahey 1979). Given these roles that soils play in shaping nutrient cycling, water availability, and microbial activity, edaphic characteristics are likely to interact with climate change factors to determine soil nutrient losses during asynchronies.

Thus, while climate change exerts top‐down pressure on nutrient cycling and the timing of biological activity, soil characteristics influence the biogeochemical response of the system from the bottom up. For example, previous work across a natural climate gradient found that variation in soil properties shaped the response of nutrient leaching to soil freezing (Groffman et al. 2011). Previously, we imposed warming and snow exclusion treatments on two different soils in a forest sapling mesocosm experiment and found that annual nutrient losses due to the climate treatments frequently varied by soil type (Juice et al. 2022). Our treatments changed climatic conditions that influence both plant phenology and microbial activity, potentially altering plant–microbe asynchronies differently across the soil type–climate treatment combinations. This possibility is supported by our finding that the ability of plant biomass to reduce leaching losses of nutrients from the mesocosms varied by soil type and climate treatment, as well as by the nutrient examined (Juice et al. 2022). If increased duration of asynchrony between plant and microbial activity amplifies C and nutrient losses, it would represent a positive feedback to climate change that could alter our predictive understanding of how climate change affects ecosystem C and nutrient retention or loss.

To investigate interactions among soil type and climate change‐driven shifts in the duration of plant‐microbe asynchronies on leaching of C and nutrients—N, P, calcium (Ca), magnesium (Mg), and aluminum (Al)—we leveraged data from our replicated climate change mesocosm experiment that imposed aboveground warming and snow exclusion treatments on two soils that differed in texture and chemical composition (Juice et al. 2022). First, we cross‐referenced soil temperature data with plant phenology data to calculate an index of plant‐microbe asynchrony represented by the number of days with soil temperatures that support microbial activity while plant metrics indicated dormancy. Then, we assessed the relationship between the duration of asynchrony and nutrient leaching from each mesocosm. We hypothesized that: (H1) warming will increase the duration of plant‐microbe asynchronies and snow exclusion will shorten it due to greater soil freezing; (H2) the effect of climate treatments on total losses of C and nutrients during plant‐microbe asynchronies will be greater from coarse soils due to their reduced capacity for water and nutrient retention; and (H3) C and nutrient losses will increase with the number of asynchrony days (i.e., greater asynchrony duration), and similarly, these losses will be greater from coarse soils. Collectively, the results of this study demonstrate that soil characteristics interact with climate treatments to modify the duration of plant‐microbe asynchrony and associated nutrient leaching, providing novel details on the mechanisms of nutrient losses under climate change.

## 2 Materials and Methods

### 2.1 Site Description and Climate Treatments

We examined how soil type, warming, and snow exclusion affected both the duration of seasonal asynchronies and nutrient losses during those asynchronies in a climate change forest sapling mesocosm experiment described in detail by Juice et al. (2022). To summarize, the field experiment was conducted at the George D. Aiken Forestry Sciences Laboratory in South Burlington, VT, USA (44°27′ N, 73°12′ W; 60 m elevation). The climate is humid continental, with a mean annual temperature of 7.3°C and average monthly temperatures ranging from −7.8°C in January to 21.3°C in July. Mean annual precipitation is 904 mm, with an average of 23% (2080 mm) falling as snow between December and March (as measured from 1950 to 2015 at the Burlington International Airport, South Burlington, VT; elevation 100 m; ~5.9 km from study site; NOAA National Weather Service 2017).

The mesocosm tanks (2.4 m diameter, 1 m soil depth, polyethylene) were initially installed in the field in 1995 as described by Beard et al. (2005). Tanks were installed belowground so that the surface of the mesocosm soil was level with the surrounding soil, with a 20 cm aboveground rim. Each mesocosm had a closed leachate drainage area with a vacuum extraction system that allowed for leachate collection. Although container effects can influence the flow of water in mesocosm experiments, it is unlikely in the current study given that: (1) the large size of the mesocosm reduces the volume of soil that is close to the container's edge; and (2) both soil types in the mesocosms had rapid infiltration rates, making them unlikely to have standing water which could move to the containers' sides in preferential flow paths (Juice et al. 2022).

Mesocosms contained one of two randomly assigned soils that were mined from unweathered glacial lake deposit substrates that differed in both physical and chemical properties (Table S1). Herein, we label the soils as “coarse” and “fine,” referring to their dominant characteristic as defined by their fine gravel contents, and to maintain consistency with prior published results (Juice et al. 2022). However, these labels represent a simplification of the suite of characteristics that defined each soil type (Table S1). Both soils had high sand contents (86.70% coarse, 87.37% fine), and the coarse soil had 57% more silt than the fine soil (10.23% coarse, 6.50% fine; Table S1). Most notably, the coarse soil contained over twice the fine gravel (44.97%; 2–5 mm diameter; Soil Science Division Staff 2017) of the “fine” soil, (17.80% fine gravel), and half the clay (3.07% coarse, 6.13% fine; Table S1). We therefore refer to the soils as coarse and fine based on their dominant difference, the fine gravel content. Fine gravel modifies various soil characteristics, including porosity, pore size distribution, and pore connectivity, as well as influencing the connectivity and interactions between the gravel and the rest of the soil (Beck‐Broichsitter et al. 2022; Chief et al. 2008; Lu et al. 2021). These characteristics then affect the soil water holding capacity, nutrient retention, and interaction of soils with nutrients in the soil water (Beck‐Broichsitter et al. 2023; Rytter 2012), making the gravel content a defining characteristic of interest in our two soil types. We therefore report soil property values on unsieved soils which contained fine gravel: the coarse soil had higher cation exchange capacity (11 meq 100 g^−1^ vs. 1 meq 100 g^−1^), higher percent C (0.698% vs. 0.325%), and higher percent N (0.045% vs. 0.031%) than the fine soil, but lower water holding capacity (9.6% vs. 14.1%), likely due to its high fine gravel content.

Mesocosms were planted in spring 2013 with four deciduous tree species with differing geographic ranges and rooting depths (Table S2): paper birch ( *Betula papyrifera* Marshall), quaking aspen ( *Populus tremuloides* Michx.), American chestnut ( *Castanea dentata* (Marshall) Borkh.), and black cherry ( *Prunus serotina* Ehrh). Twenty saplings of each species were equally spaced and randomly distributed within each mesocosm. Summer 2013 represented the sapling establishment period. In fall 2013, we simulated natural forest floor conditions by adding a homogenized mix of air‐dried and chopped leaves (to 2.2 cm depth) of the four species that we collected in litter traps from local mature trees. During the experiment, all plants other than the saplings were weeded and left on the mesocosm soil surface.

In December 2013, we initiated climate treatments based on low CO_2_ emissions scenario model projections for the northeastern United States in the year 2100 (Frumhoff et al. 2007). Treatments consisted of control, infrared (IR) warming of 2°C above ambient, and snow exclusion at the beginning of winter. They were randomly imposed on the two soil types (fine and coarse) in a factorial design across 24 mesocosms, resulting in four replicates of each soil–climate treatment combination (fine control, fine warming, fine snow exclusion, coarse control, coarse warming, coarse snow exclusion).

For the warming treatment, we suspended 4 ceramic IR warming elements (Kimball et al. 2008; Mor Electric Heating, Comstock Park, MI, USA, FTE‐1000‐240‐0‐L6‐WH‐0240 V 1000 W), encased in aluminum extrusion reflectors (Mor Electric Heating) and inverted aluminum gutters, around each mesocosm's perimeter at 1.5 m height (45° angle) on 5 cm diameter galvanized steel posts that were located outside the mesocosm tanks. Surface temperature in the center of IR‐warmed and control mesocosms was measured with radiometers (Apogee Instruments, Logan, UT; SI‐111) controlled by a CR1000 datalogger (Campbell Scientific, Logan, UT). Radiometers were used to maintain IR‐warmed mesocosms 2°C warmer than their paired control tanks. To standardize infrastructure effects across treatments, control and snow exclusion mesocosms had identical, nonfunctional heater assemblies. To minimize wind interference from December to June, we enclosed each mesocosm perimeter within 0.6 m tall clear plastic sheeting.

We excluded snow by covering each snow exclusion mesocosm with tarps during snow events for 6 weeks following the first snowstorm of the year. Snow exclusion began on December 14, 2013 and December 9, 2014 for winters 2013/2014 and 2014/2015, respectively. Prior to the initiation of snow exclusion, we allowed two inches of snow to accumulate to maintain low albedo across treatments, thereby avoiding any warming effect of bare soil (Groffman et al. 2001). For more detail on mesocosms and experimental design, see Juice et al. (2022).

### 2.2 Sapling Phenology Measurements

We assessed sapling spring phenology with a numerical rating system modified from West and Wein (1971; Table S2). In spring 2014, we assessed bud and leaf development of all saplings in each mesocosm weekly until leaves were fully expanded. Because the saplings of each species followed a similar phenological trajectory, in spring 2015, the same protocol was used but on a subset of four saplings per species per mesocosm. In all cases, each plant's most advanced phenological stage and the percentage of the plant that had reached that stage were identified. To relate plant phenology to the bulk movement of water and nutrients from the mesocosm, we calculated the mean plant phenological stage for each mesocosm by date by averaging the values for all the plants together, rather than examining species‐level responses, which are outside the scope of this study.

Fall phenology was measured in 2014 by digitally assessing vegetation color. We could not assess fall phenology in 2013 because climate treatments were initiated in December that year. From the onset of fall until leaf drop in 2014, weekly photos were taken of each mesocosm from the same location and angle at the four cardinal directions (4 photos mesocosm^−1^ day^−1^). Using ImageJ software (Schneider et al. 2012), we identified pixel color (green, yellow or red) according to unique color spectral ranges. The hue, saturation, and brightness (HSB) color space was used to quantify green (H: 50–141, S: 0–255, B: 0–255) and yellow (H: 30–49, S: 0–255, B: 110–255) pixels using the color thresholder function in ImageJ. The L*a*b* color space model function was used to define the red color class to solve the complication caused by the presence of red instruments in the images. This model represents colors in three‐dimensional space, with one axis for luminance (*L*) and two for colors (*a* and *b*). The threshold ranges used for red were *L* (122–209), *a* (126–255), and *b* (163–201). For each mesocosm, we calculated the mean for each color on each date. Green, yellow, and red pixels represented all the foliage in each mesocosm. Green pixels represented potentially active foliage (i.e., photosynthesizing and transpiring).

We analyzed the average phenology curve for each mesocosm to identify when plant activity declined in the fall, based on the curve of percent green pixels from the photographic analysis, and resumed in the spring, based on the curve of the phenology ratings resulting from the visual measurements. In the fall, declining canopy photosynthetic capacity is indicated by a turning point in the phenology curve called the vegetation downturn day (Gu et al. 2009). For fall 2014, we fit the curve of the percent green pixels for each mesocosm (as in Klosterman et al. 2014) and the downturn day was identified using the greenProcess function in the R phenopix package (Filippa et al. 2017). At the end of the rapid expansion phase of spring leaf out, the stabilization date (Gu et al. 2009) marks the end of the period of possible asynchronies as plants resume significant activity levels. We identified the stabilization date for each mesocosm by fitting the spring phenology curve (as in Gu et al. 2009) using the greenProcess function in the R phenopix package (Filippa et al. 2017).

### 2.3 Soil Temperature Measurements

Average soil temperature was recorded at 5‐min intervals (CR1000 datalogger) in each mesocosm at 5 cm depth using type T thermocouples (Omega Engineering Inc., Stamford, CT). The five‐minute means were used to calculate average hourly soil temperatures. Because soil activity responds quickly to temperature, we subset the data to daylight hours to capture any possible activity during warm days even if the soil cooled below 4°C at night. Determination of daylight hours was based on sunrise and sunset data from the National Oceanic and Atmospheric Agency (NOAA Solar Calculator, https://gml.noaa.gov/grad/solcalc/).

Soil temperature data were analyzed to determine the day of year the soil in each mesocosm cooled below 4°C in the fall, and warmed above 4°C in the spring (i.e., the “spring trigger” *sensu* Groffman et al. (2012)). The total number of days that soils were above 4°C during plant dormancy and between mesocosm leachate extraction dates was also calculated as the total plant–microbe asynchrony (see next section).

### 2.4 Identifying Asynchronies

Soil temperature data were cross‐referenced with fall and spring phenology data to identify the potential period of plant‐microbe asynchronies when soil temperature allowed microbial activity, but plant metrics indicated dormancy. The potential for asynchronies to occur exists anytime between the diminution of plant activity in autumn (plant downturn day) and leaf expansion in spring (plant stabilization date). However, it was not feasible to align our mesocosm leachate collection with the plant downturn day and the plant stabilization date, both of which varied by mesocosm and were unknown until after analysis of the mesocosm‐level phenology curves. To accurately assess the relationship between asynchrony duration and associated soil nutrient leaching, we had to constrain our analysis of the asynchrony duration to the period that aligned with the mesocosm leachate collection (see leachate collection methods below and Figure S1; winter 1: 2/15/2014–5/8/2014; winter 2: 11/10/2014–5/7/2015). Although constraining the period of potential asynchronies to the dates between leachate collection may result in an underestimate of the total asynchrony length (and, indeed, total asynchrony nutrient losses), it is necessary so that the leachate chemistry data are compared to the number of asynchrony days associated with the leaching and not a longer asynchrony period as defined by the plant phenology metrics. Although belowground biological processes occur under snow (Brooks et al. 2011), the rate is relatively low below 4°C, the point at which rapid biological activity is thought to begin (Groffman et al. 2012). Therefore, we considered mean soil temperatures during daylight hours that were ≥ 4°C at 5 cm depth to be indicative of potential soil activity. We then counted the number of days with daylight mean soil temperature ≥ 4°C at 5 cm depth between mesocosm leachate extraction dates (during plant dormancy) to calculate the duration of plant‐microbe asynchrony for each mesocosm.

To ensure that the trends in asynchrony duration observed between leachate extraction dates were representative of the trends occurring between the plant phenological metrics, we additionally counted the number of days with daylight mean soil temperature ≥ 4°C at 5 cm depth between the fall downturn day and the spring stabilization date in Year 2 (Figure S2). We were unable to conduct this analysis with Year 1 data, because we did not initiate experimental climate treatments until December 2013, and therefore do not have phenology data for Fall 2013. However, consistent with our analysis of asynchrony duration between mesocosm leachate collections, in Year 2 we found that asynchrony duration was significantly longer in the warmed mesocosms (Figure S2; ${Χ}_{2}^{2}$ = 20.1, *p* < 0.0001, *R* ^*2*^ = 0.59), providing support for the validity of our findings on climate treatment effects on asynchrony duration constrained to the leachate collection period.

### 2.5 Leachate Collection and Analysis

Leachate was extracted periodically during snow‐free periods when the water rose to the height of the leachate drainage area, as assessed by inserting a measurement rod into the center tube to the bottom of the mesocosm (Juice et al. 2022). The volume of water removed was measured by a totalizer attached to a pump, and a sample was collected, filtered using 0.45 μm nylon filters (Fisher Scientific, Hampton, NH, cat. no. 09‐719‐008), and frozen until analysis to prevent transformation of nutrients (Menchyk et al. 2014).

Leachate samples were analyzed for (1) Ammonium (${\mathrm{NH}}_{4}^{+}$) using a salicylate method modified from Weatherburn (1967) and analyzed with a Synergy HT Microplate Reader (BioTek Instruments, Winooski, VT), (2) nitrate‐nitrite (${\mathrm{NO}}_{3}^{-}$ + ${\mathrm{NO}}_{2}^{-}$; hereafter referred to as ${\mathrm{NO}}_{3}^{-}$) and phosphate (${\mathrm{PO}}_{4}^{3-}$) using colorimetric methods and a Lachat QuikChem 8000 flow‐injection analyzer (Lachat Instruments, Hach Company, Loveland, CO), (3) dissolved organic C (DOC) and total dissolved N (TDN) by sample combustion followed by infrared gas analysis and chemiluminescence for DOC and TDN, respectively, using a Total Organic C Analyzer (Shimadzu TOC‐L with TNM‐L, Columbia, MD), and (4) Ca, Mg, and Al by inductively coupled plasma atomic emission spectroscopy (ICP‐AES) on an Optima 3000DV (Perkin Elmer Inc., Boston, MA). Nutrient concentration was multiplied by leachate volume to calculate total losses on each sampling date. Losses were summed across leachate extraction dates that occurred during the potential asynchrony period for each year to examine the impact of climate treatments, soil type, and asynchrony duration on total loss of C and other nutrients.

### 2.6 Statistical Analyses

All statistical analyses were performed in R (R Core Team 2017). We determined the responses of asynchrony duration (in days) and leachate loss of C (DOC) and other nutrients (TDN, ${\mathrm{NO}}_{3}^{-}$, ${\mathrm{NH}}_{4}^{+}$, ${\mathrm{PO}}_{4}^{3-}$, Ca, Mg, Al) during asynchronies to soil and climate treatments using generalized least squares (gls) models (nlme package; Pinheiro et al. 2017). Asynchrony duration and climate treatments were collinear (variance inflation factor, VIF > 10), so leachate losses of C and nutrients were examined as a function of (i) soil and climate treatment and (ii) soil and asynchrony duration in separate models. Significance of model terms was determined with type 3 (partial) Analysis of Deviance models (car package; Fox and Weisberg 2011). For all models, assumptions of constant variance and normality were assessed by inspection of residuals. When necessary, variance structures were constructed for categorical and continuous variables using the varIdent and varPower functions, respectively (nlme package; Pinheiro et al. 2017), and power transformations were applied to non‐normal data. Results were considered significant at *p* < 0.05. Unless otherwise noted, reported values are means ± standard errors.

## 3 Results

### 3.1 Plant‐Microbe Asynchrony Duration, Soil Temperature, and Plant Phenology

**FIGURE 1:**
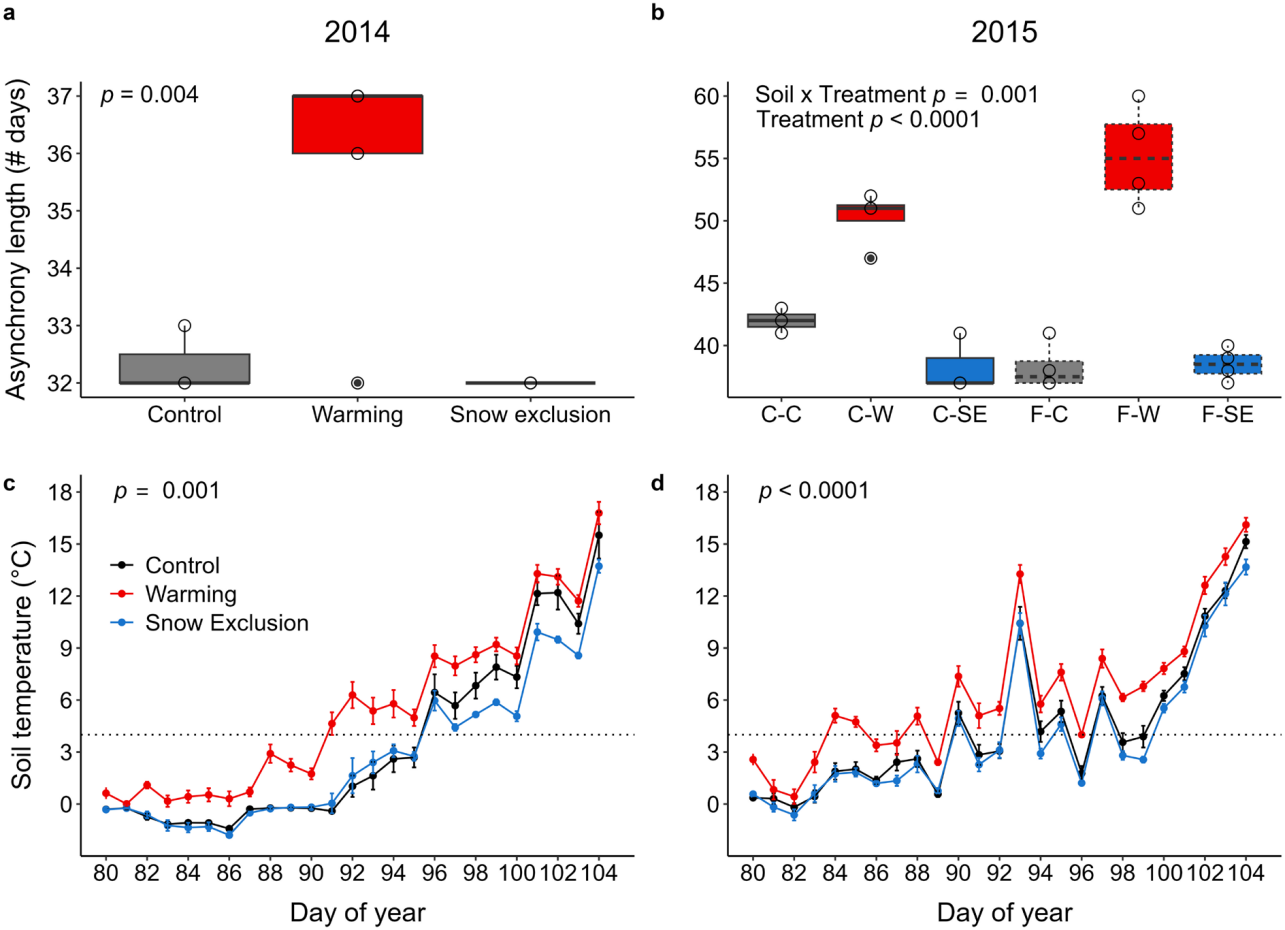
Total asynchrony duration (a) and (b) and springtime soil temperatures (c) and (d) measured across 2 years in an in‐field forest sapling mesocosm experiment in South Burlington, VT. Asynchrony duration in (a) 2014 and (b) 2015 are shown by the significant model terms for each year. We calculated asynchrony duration as the number of days with daytime soil temperatures ≥ 4°C at 5 cm depth while plants were dormant between mesocosm leachate collection dates. Open circles represent data points and closed circles represent outliers; note the varying y‐axis limits in panels a and b. X‐axis codes in panel b are C (coarse soil, solid lines) or F (fine soil, dashed lines) followed by treatment (C = control (gray), W = warming (red), SE = snow exclusion (blue)). Springtime soil temperatures in (c) 2014 and (d) 2015 were measured at 5 cm depth. The dotted horizontal line in panels c and d marks 4°C, the soil temperature at which rapid biological activity is thought to begin (Groffman et al. 2012).

Warming increased the duration of plant‐microbe asynchronies, and the effect of snow removal varied among years and soil types. In 2014, warming increased asynchrony duration by an average of three and a half days over controls, which were similar to snow exclusion (Figure 1a, ${Χ}_{2}^{2}$ = 11.1, *p* = 0.004, *R* ^*2*^ = 0.75). In 2015, treatment effects (${Χ}_{2}^{2}$ = 118.7, *p* < 0.0001, *R* ^*2*^ = 0.92) varied across soils (Figure 1b, soil × treatment interaction; ${Χ}_{2}^{2}$ = 13.4, *p* = 0.001). On coarse soils, warming increased and snow exclusion decreased asynchrony duration by an average of eight and four days, respectively. On fine soils, warming increased asynchrony duration by an average of 17 days over controls, which were comparable to snow exclusion. The calculated asynchrony duration is shorter for the first year of the study because climate treatments were initiated in December that year, causing the fall asynchrony days to be excluded from our calculations.

The warming treatment significantly accelerated soils reaching 4°C in spring, but climate treatments had no effect on the timing of soils cooling below 4°C in the fall (Figure S3). In spring 2014, warmed mesocosm soils reached daily mean temperature of 4°C (during daylight hours) four days earlier than control or snow exclusion soils (Figure 1c; ${Χ}_{2}^{2}$ = 13.9, *p* = 0.001, *R* ^*2*^ = 0.77). The effect was greater in 2015, when warmed mesocosm soils reached 4°C 7 days before control or snow exclusion soils (Figure 1d, ${Χ}_{2}^{2}$ = 37.8, *p* < 0.0001, *R* ^*2*^ = 0.80).

Conversely, climate treatments had significant effects on the timing of plant activity in the fall, but not in the spring (Figure S4). Specifically, in fall 2014 plants growing in warming and snow exclusion mesocosms reached their downturn day one and two days later than control plants, respectively (${Χ}_{2}^{2}$ = 6.1, *p* = 0.047, *R* ^*2*^ = 0.36).

### 3.2 Carbon and Nutrient Loss During Asynchronies

**FIGURE 2:**
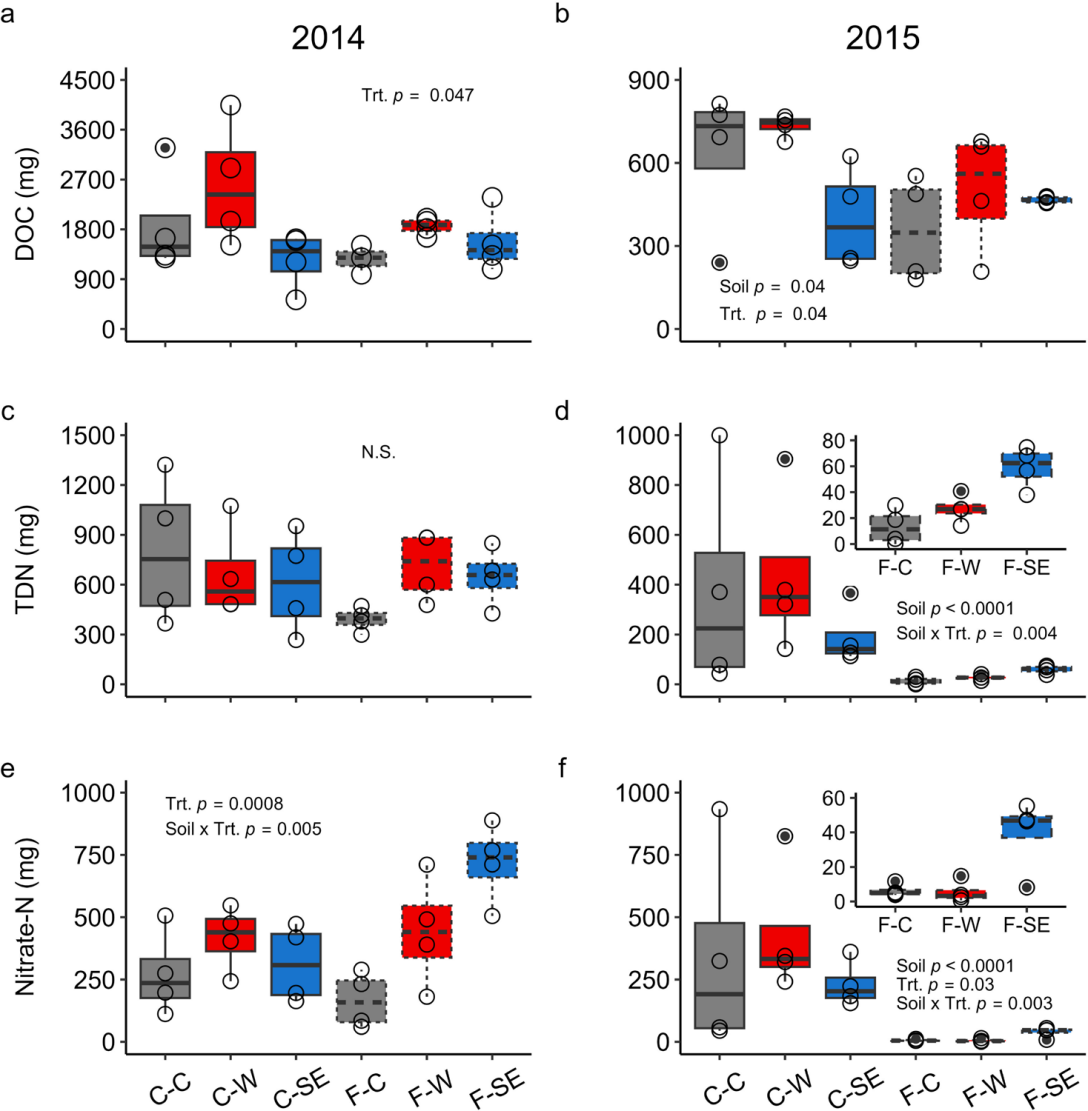
Nutrient leachate loss as related to soil type and climate treatment during plant‐microbe asynchronies in an in‐field forest sapling mesocosm experiment in South Burlington, VT: Dissolved organic C (DOC) loss in (a) 2014 and (b) 2015; total dissolved N (TDN) loss in (c) 2014 and (d) 2015; and nitrate‐N loss in (e) 2014 and (f) 2015. Significant model terms and *p*‐values are annotated on each panel, with treatment abbreviated as “Trt” and “N.S” indicating not significant. Open circles represent data points and closed circles represent outliers; note the varying y‐axis limits in panels a and b. X‐axis codes in are C (coarse soil, solid lines) or F (fine soil, dashed lines) followed by treatment (C = control (gray), W = warming (red), SE = snow exclusion (blue)). See Table 1 for associated analysis of deviance results.

During asynchronies, DOC loss was significantly impacted by climate treatment, with no significant interactions with soil type (Table 1, Figure 2a,b). Overall, warming increased DOC losses in both years (with a larger effect in 2014), while snow exclusion decreased losses but only in 2015 (Figure 2a,b). Warming in 2014 increased asynchrony DOC losses by 50% (2230 mg ± 298) relative to controls (1493 mg ± 277), which had similar losses as from snow exclusion (1410 mg ± 187; Figure 2a). In 2015, warmed mesocosm DOC losses (618 mg ± 68) were 25% higher than control losses (494 mg ± 91) and losses from snow exclusion (434 mg ± 44) were 12% lower than control losses. Furthermore, soil type only had a significant impact on DOC losses in 2015 when losses were 33% greater from coarse (588 mg ± 64) than fine soils (442 mg ± 47; Figure 2b).

**TABLE 1:** Analysis of deviance results (*Χ* ^*2*^ *,* degrees of freedom (df), and *p* values) for soil water leachate carbon and nutrients lost as a function of soil × climate treatment during plant‐microbe asynchronies across 2 years in an in‐field forest sapling mesocosm experiment in South Burlington, VT. We calculated asynchrony duration as the number of days with daytime soil temperatures ≥ 4°C at 5 cm depth while plants were dormant between mesocosm leachate collection dates.

Variable (df)		2014				2015			
		Soil (1)	Trt (2)	Soil × Trt (2)	*R* ^2^	Soil (1)	Trt (2)	Soil × Trt (2)	*R* ^2^
DOC	*Χ* ^*2*^	2.0	6.1	4.2	0.40	4.1	6.5	5.8	0.42
	*p*	0.16	0.047*	0.12		0.04*	0.04*	0.06	
TDN	*Χ* ^*2*^	0.6	1.4	3.7	0.24	34.5	3.2	10.8	0.72
	*p*	0.46	0.49	0.15		< 0.0001*	0.20	0.004*	
${\mathrm{NO}}_{3}^{-}$	*Χ* ^*2*^	2.7	14.2	10.8	0.61	79.2	6.9	11.9	0.84
	*p*	0.10	0.0008*	0.005*		< 0.0001*	0.03*	0.003*	
${\mathrm{NH}}_{4}^{+}$	*Χ* ^*2*^	0.1	0.2	3.6	0.16	2.0	0.1	1.1	0.15
	*p*	0.75	0.9	0.17		0.16	0.94	0.58	
${\mathrm{PO}}_{4}^{3-}$	*Χ* ^*2*^	13.0	1.5	0.2	0.45	15.2	5.9	3.5	0.55
	*p*	0.0003*	0.48	0.91		< 0.0001*	0.05	0.17	
Ca	*Χ* ^*2*^	32.6	0.9	3.7	0.67	93.8	32.7	3.1	0.87
	*p*	< 0.0001*	0.65	0.16		< 0.0001*	< 0.0001*	0.22	
Mg	*Χ* ^*2*^	0.2	2.9	7.1	0.28	40.5	42.8	10.2	0.81
	*p*	0.67	0.23	0.03*		< 0.0001*	< 0.0001*	0.006*	
Al	*Χ* ^*2*^	3.9	1.7	5.2	0.36	0.8	3.4	7.7	0.24
	*p*	0.049*	0.43	0.07		0.39	0.18	0.02*	

Abbreviations: ${\mathrm{NH}}_{4}^{+}$, ammonium; ${\mathrm{NO}}_{3}^{-}$, nitrate; ${\mathrm{PO}}_{4}^{3-}$, phosphate; Al, aluminum; Ca, calcium; DOC, dissolved organic carbon; Mg, magnesium; TDN, total dissolved nitrogen.

* *p* < 0.05.

Nitrogen losses during asynchronies were significantly altered by soil type and climate treatment (Table 1, Figure 2c–f). While asynchrony TDN loss was not significantly related to soil or climate treatment in 2014 (Figure 2c), in 2015, asynchrony losses of TDN from coarse soils (333 mg ± 91) were 10 times greater than from fine soils (33 mg ± 7; significant soil effect, Figure 2d). Furthermore, the response to treatment varied by soil type in 2015 (Figure 2d), such that in coarse soils, neither warmed losses (437 mg ± 164) nor snow exclusion losses (190 mg ± 59) varied significantly from control (373 mg ± 221). Conversely, on fine soils, TDN losses were 350% greater from snow exclusion (59 mg ± 8) and 108% greater from warmed (27 mg ± 5) mesocosms relative to controls (13 mg ± 7).

Unlike TDN, ${\mathrm{NO}}_{3}^{-}$ significantly varied by soil and treatment both years of the study (Figure 2e,f). In 2014 (Figure 2e), the response of ${\mathrm{NO}}_{3}^{-}$ loss to treatment varied by soil type. On coarse soils, ${\mathrm{NO}}_{3}^{-}$ losses were comparable across treatments (control: 273 mg ± 85; warmed: 418 mg ± 65; snow exclusion: 313 mg ± 78). However, on fine soils, snow exclusion resulted in a 330% increase in ${\mathrm{NO}}_{3}^{-}$ losses (719 mg ± 80) and warming resulted in a 166% increase in ${\mathrm{NO}}_{3}^{-}$ loss (444 mg ± 110) compared to controls (167 mg ± 56). In 2015 (Figure 2f), ${\mathrm{NO}}_{3}^{-}$ loss from coarse soils was nearly 20 times higher (335 mg ± 80) than from fine soils (17 mg ± 6) and the effect of treatment varied by soil type. On coarse soils, ${\mathrm{NO}}_{3}^{-}$ losses from warming (433 mg ± 133) and snow exclusion (231 mg ± 46) were comparable to controls (340 mg ± 208). On fine soils, snow exclusion (39 mg ± 11) elevated ${\mathrm{NO}}_{3}^{-}$ losses by 550% compared to controls (6 mg ± 2), which were similar to warmed mesocosms (5 mg ± 3). Ammonium losses did not vary significantly by soil or treatment either year of the study.

In contrast to N losses, asynchrony losses of ${\mathrm{PO}}_{4}^{3-}$ were only impacted by soil type, with no effect of climate treatments. Losses of ${\mathrm{PO}}_{4}^{3-}$ were significantly higher from fine (2014: 18 mg ± 1; 2015: 20 mg ± 2) than coarse soils (2014: 13 mg ± 1; 2015: 12 mg ± 1) both years, with no significant effect of climate treatment. This is despite the coarse soil having higher total P than the fine soil (Table S1).

Cation losses varied significantly by soil and climate treatment, with more climate treatment effects in 2015 (Table 1). In both years, asynchrony Ca loss was over 75% higher from coarse (2014: 35 g ± 3; 2015: 23 g ± 1) than fine soils (2014: 17 g ± 1; 2015: 13 g ± 1). Climate treatments did not impact Ca leached in 2014, but in 2015, losses from snow exclusion (16 g ± 2) were reduced 24% compared to controls (21 g ± 2), which had similar Ca losses as from warmed mesocosms (18 g ± 2). Similar to Ca, Al loss in 2014 was 18% higher from coarse (311 mg ± 30) than fine (263 mg ± 21) soils. In 2015, soil type interacted with climate treatment to impact Al losses. On coarse soils, snow exclusion (141 mg ± 8) Al losses were 21% higher than control (117 mg ± 14) losses, which were similar to warmed (130 mg ± 10) treatments. On fine soils, Al losses were similar in all three climate treatments (control: 139 mg ± 28; warming: 163 mg ± 5; snow exclusion: 109 mg ± 20). The effect of climate treatment on asynchrony Mg loss varied by soil type during both years of the experiment. In 2014, coarse soil losses were comparable across treatments (control: 4.6 g ± 0.5; warmed: 4.1 g ± 0.3; snow exclusion: 4.2 g ± 0.9), but on fine soils, warming (4.8 g ± 1.2) and snow exclusion (4.7 g ± 0.4) increased Mg losses by 80% relative to control (2.7 g ± 0.4). In 2015, Mg losses from coarse soils experiencing warming (2.6 g ± 0.1) or snow exclusion (2.5 g ± 0.04) were reduced 24% compared to control (3.4 g ± 0.2). Conversely, on fine soils, warming (2.5 g ± 0.3) elevated Mg losses by 25% compared to control (2.0 g ± 0.1), while snow exclusion (1.4 g ± 0.1) decreased Mg losses by 30%. Also in 2015, Mg losses from coarse soil (2.8 g ± 0.1) were 40% greater than from fine soil (2.0 g ± 0.2; significant soil effect).

### 3.3 Carbon and Nutrient Losses as Related to Asynchrony Duration

**FIGURE 3:**
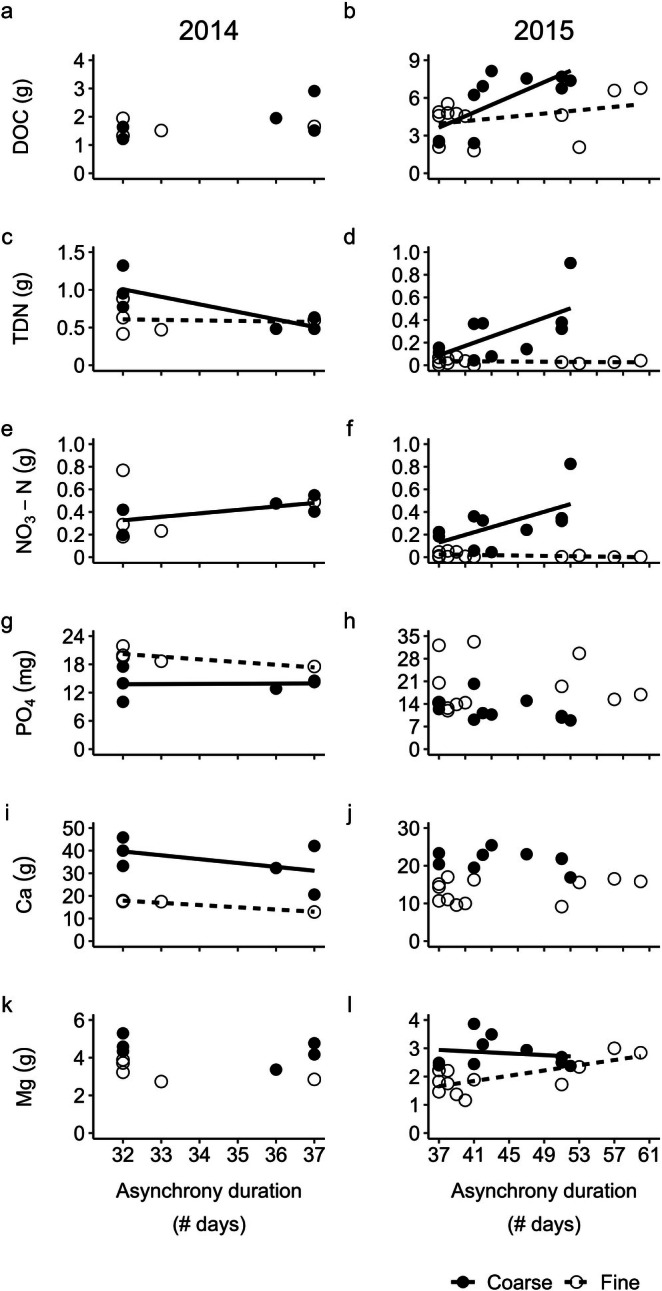
Nutrient leachate loss as related to asynchrony duration and soil type in an in‐field forest sapling mesocosm experiment in South Burlington, VT in 2014 (left column) and 2015 (right column): (a) and (b) dissolved organic carbon (DOC); (c) and (d) total dissolved nitrogen (TDN); (e) and (f) nitrate‐N (${\mathrm{NO}}_{3}^{-}$‐ N); (g) and (h) phosphate (${\mathrm{PO}}_{4}^{3-}$); (i) and (j) calcium (Ca); (k) and (l) magnesium (Mg). Asynchrony duration is the number of days with daytime soil temperatures ≥ 4°C at 5 cm depth while plants were dormant between mesocosm leachate collection dates. Regression lines show significant relationships. One regression line indicates losses varied significantly with asynchrony duration, and two regression lines indicate the relationship varied between coarse (closed circles and solid line) and fine (open circles, dashed lines) soils. See Table 2 for associated analysis of deviance results. Note different units for different nutrients in the axis titles.

Nutrients leached during asynchronies showed significant relationships to soil and duration of asynchrony, and their interaction (Table 2, Figure 3). DOC loss in 2015 (Table 2, Figure 3b) increased significantly with asynchrony duration, and the effect was greater on coarse soil (soil*asynchrony duration interaction; coarse: 30 mg DOC/day, fine: 7 mg DOC/day). The relationship between nitrogen losses (TDN and ${\mathrm{NO}}_{3}^{-}$) and asynchrony duration varied over time and between soil types. TDN loss from coarse soils decreased significantly with asynchrony duration in 2014, and increased significantly with asynchrony duration in 2015 (Table 2, Figure 3c,d; 2014: −91 mg TDN/day, 2015: 30 mg TDN/day). Increased ${\mathrm{NO}}_{3}^{-}$ losses in 2014 were associated with longer asynchronies across soil types (asynchrony duration effect, Table 2, Figure 3e), with 0.1 mg ${\mathrm{NO}}_{3}^{-}$ loss per day associated with each additional day of asynchrony. The following year, the effect of asynchrony duration on ${\mathrm{NO}}_{3}^{-}$ loss varied by soil. Although the loss rates per day were significantly different across soil types, the amounts lost were relatively small (Table 2, Figure 3f; coarse: 0.07 mg ${\mathrm{NO}}_{3}^{-}$/day, fine: −0.09 mg ${\mathrm{NO}}_{3}^{-}$/day). Most notably, on coarse soils asynchrony duration had a positive relationship with ${\mathrm{NO}}_{3}^{-}$ loss, while on fine soils that relationship was negative.

**TABLE 2:** Analysis of deviance results (*Χ* ^*2*^ *,* degrees of freedom (df), and *p* values) for soil water leachate carbon and nutrients lost as a function of soil × asynchrony duration across 2 years in an in‐field forest sapling mesocosm experiment in South Burlington, VT. We calculated asynchrony duration as the number of days with daytime soil temperatures ≥ 4°C at 5 cm depth while plants were dormant between mesocosm leachate collection dates.

Variable (df)		2014				2015			
		Soil (1)	Duration (2)	Soil × duration (2)	*R* ^2^	Soil (1)	Duration (2)	Soil × duration (2)	*R* ^2^
DOC	*Χ* ^*2*^	0.0	0.6	0.0	0.33	3.2	10.6	4.3	0.45
	*p*	0.94	0.43	0.96		0.07	0.001*	0.04*	
TDN	*Χ* ^*2*^	4.0	3.9	3.9	0.52	3.3	4.9	5.2	0.62
	*p*	0.047*	0.049*	0.0497*		0.07	0.03*	0.02*	
${\mathrm{NO}}_{3}^{-}$	*Χ* ^*2*^	0.04	5.0	0.04	0.28	2.1	0.01	7.1	0.79
	*p*	0.84	0.03*	0.85		0.15	0.75	0.008*	
${\mathrm{NH}}_{4}^{+}$	*Χ* ^*2*^	0.9	0.8	0.9	0.31	0.5	0.1	0.4	0.05
	*p*	0.34	0.36	0.36		0.47	0.77	0.51	
${\mathrm{PO}}_{4}^{3-}$	*Χ* ^*2*^	5.1	0.0	4.2	0.75	0.6	1.4	1.9	0.39
	*p*	0.02*	1.0	0.04*		0.45	0.23	0.17	
Ca	*Χ* ^*2*^	6.7	30.8	11.3	0.93	5.9	0.3	2.6	0.71
	*p*	0.009*	< 0.0001*	0.0008*		0.02*	0.60	0.11	
Mg	*Χ* ^*2*^	0.0	2.7	0.0	0.65	9.2	0.5	6.6	0.62
	*p*	0.97	0.10	0.83		0.002*	0.46	0.01*	
Al	*Χ* ^*2*^	0.0	0.1	0.1	0.14	0.1	3.1	0.2	0.16
	*p*	0.8	0.7	0.8		0.79	0.08	0.65	

Abbreviations: ${\mathrm{NH}}_{4}^{+}$, ammonium; ${\mathrm{NO}}_{3}^{-}$, nitrate; ${\mathrm{PO}}_{4}^{3-}$, phosphate; Al, aluminum; Ca, calcium; DOC, dissolved organic carbon; Mg, magnesium; TDN, total dissolved nitrogen.

* *p* < 0.05.

This trend of the direction of the relationship between nutrient losses and asynchrony duration varying across soil type was true of ${\mathrm{PO}}_{4}^{3-}$ losses in 2014 as well (Table 2, Figure 3g; coarse: 0.46 mg ${\mathrm{PO}}_{4}^{3-}$/day, fine: −0.48 mg ${\mathrm{PO}}_{4}^{3-}$/day), and of Mg losses in 2015 (Figure 3l; coarse: −29 mg Mg/day, fine: 52 mg Mg/day). Even though the relationship between asynchrony duration and Ca loss in 2014 varied significantly across soil type, losses associated with asynchrony duration from both soil types were less than 0.00001 mg Ca/day, and therefore unlikely to be ecologically relevant. In 2015, asynchrony losses of Ca were higher from coarse soil than fine soil, but showed no relationship to duration of asynchrony (soil effect, Table 2, Figure 3j). Ammonium and Al losses did not vary by soil type or asynchrony duration either year of the study (Table 2, Figure S5).

## 4 Discussion

Our results show that climate change can alter the timing of plant phenology and soil microbial activity, acting to increase (in the case of warming) or decrease (in the case of snow exclusion) the duration of periods with microbial activity during plant dormancy. Further, our results suggest that the asynchrony period and its duration are important for determining annual C and N losses. When compared to total annual losses from the same experiment (Juice et al. 2022), asynchrony C and N losses ranged from 60% to 80% of total annual losses. Moreover, the impact of climate treatments on soil carbon and nutrient losses during plant‐microbe asynchronies varied significantly by soil type, demonstrating the importance of edaphic characteristics such as gravel content, water holding capacity, and cation exchange capacity in determining the occurrence and magnitude of nutrient losses under climate change. Longer periods of asynchrony also often correlated with increased nutrient leaching, indicating that phenological perturbations due to climate change could reduce overall ecosystem nutrient retention and potentially feed back to reduced plant productivity. Overall, our results provide evidence that interactions among climate treatments and soil properties are extremely prevalent and an important determinant of the magnitude of climate change effects on plant‐microbe synchrony and ecosystem biogeochemistry.

*Warming increased and soil freezing at times decreased asynchronies*.

As hypothesized, warming increased plant‐microbe asynchrony duration, coinciding with findings from long‐term ecological studies (Contosta et al. 2017; Groffman et al. 2012). The longer asynchronies were mainly due to the accelerated ramp‐up of springtime soil temperatures in warmed mesocosms, since warming had only a slight effect on fall plant activity and no effect on the day of fall soil cooling or springtime plant phenology. In fact, the increase in asynchrony duration in warmed mesocosms in 2014 exactly coincides with the acceleration of warmed mesocosm soils reaching 4°C as compared to control soils (~4 days). However, the muted response of plant phenology to warming in the mesocosms contrasts with previous studies that have found phenology to be advancing in the spring (Thompson and Clark 2008) and delayed in the fall (Jeong et al. 2011) due to climate warming, albeit with heterogeneity (Dragoni and Rahman 2012). The lack of response by saplings in the mesocosm could be related to their life stage, since juvenile tree phenology differs from mature trees (Augspurger and Bartlett 2003). Alternatively, the open conditions of the mesocosm, which approximate a forest gap with no overstory trees, have also been found to change leaf longevity in saplings, with effects varying by species (Augspurger and Bartlett 2003). Because we were interested in the bulk movement of water and nutrients through the plant biomass, we grouped the sapling species together for analysis. This additionally could have obscured species‐level differences in the sapling response to warming treatments.

Although warming consistently increased the duration of plant‐microbe asynchronies, the magnitude of the effect varied across soil types and years. In the first year of the study, warming increased asynchronies equally on both soils. In contrast, in the second year, asynchronies on warmed fine soils were twice as long as on warmed coarse soils (17 days on fine vs. 8 days on coarse soil). Because there were no soil differences in the timing of soil cooling in the fall, soil warming in the spring, or plant phenology, we conclude that the difference between the soils occurred over the course of the entire period of plant dormancy, including winter thaws. In our experiment, climatic variables including snow depth, soil frost depth, and soil temperature all varied with climate treatments but not with soil types (Juice et al. 2022). Instead, it appears that soil characteristics affected the nature of soil freezing in such a way that altered thaw dynamics and the timing of soil warming to 4°C to support rapid microbial activity in the upper soil profile. For example, the hardness of soil frost can vary by soil texture and moisture (e.g., unsaturated vs. concrete frost; Gray and Granger 1986), with saturated concrete frosts thawing more slowly than unsaturated frosts. Between our two soil types, we would expect the fine soil to freeze harder and thaw more slowly due to its finer texture, higher moisture content (over twice the soil moisture; Juice et al. 2022), and greater plant biomass (24% more total plant biomass in fine vs. coarse soils measured at the end of the experiment; Juice et al. 2022). Despite this, our results show that soil temperatures rose faster in the fine soil than in the coarse soil throughout the second year of the experiment (Figure S6), resulting in greater duration of asynchrony under warming of fine soils.

Finally, as hypothesized, snow exclusion shortened asynchrony duration, but the effect varied across years and soils. In the second year, plant‐microbe asynchronies in the coarse soil snow exclusion treatment were, on average, 4 days shorter than control. This may be partially explained by fall plant phenology, which was delayed by 2 days in the snow exclusion treatment. However, fall plant phenology did not vary by soil type. It is therefore likely that the dynamics leading to shortened plant‐microbe asynchronies under snow exclusion are similar to those that increased the asynchrony on fine warmed soils. Namely, the characteristics of the two soil types altered the nature of the soil frost that occurred, increasing the time to thaw and warm under snow exclusion on coarse, but not fine, soils. This increased time to warm would reduce the overall number of days with warm soil temperatures during plant dormancy on coarse soils experiencing snow exclusion. In the case of both warming and snow exclusion, soil properties therefore modified the effect of the climatic changes on the soil environment and its ability to support biological activity, with important consequences for ecosystem‐level retention of carbon and nutrients.

*Climate treatments elevated asynchrony losses more from fine than coarse soils*.

Contrary to our hypothesis, climate treatments generally elevated C and nutrient losses from fine soils more than from coarse soils during plant‐microbe asynchronies. This was true for losses of TDN, ${\mathrm{NO}}_{3}^{-}$, Mg, and Al during one or both years. Although coarse soils on the whole experienced greater N losses than fine soils in 2015, the pattern of climate treatments elevating N losses from fine but not coarse soils remained. TDN and ${\mathrm{NO}}_{3}^{-}$ followed similar patterns, with snow exclusion and warming greatly elevating N losses from fine soils but not coarse soils, with some variation across time. Increased ${\mathrm{NO}}_{3}^{-}$ loss following soil freezing is well‐documented (Campbell et al. 2014; Mitchell et al. 1996), although with variability (Groffman et al. 2011; Judd et al. 2011), and has been attributed to root mortality (Tierney et al. 2001) and decreased root nutrient uptake (Campbell et al. 2014). These N leaching results from the asynchrony period presented here coincide with previously published data from the full growing season (Juice et al. 2022). In that paper, we concluded that soil properties could explain ${\mathrm{NO}}_{3}^{-}$ loss following soil freezing in addition to the proposed root mechanisms (Juice et al. 2022). That conclusion is further strengthened by the current findings, in which significant variation in N leaching across soil types occurred during periods without plant activity, hence eliminating reduced plant nutrient uptake or root effects as possible causes of the observed increase in leaching. Warming also intermittently elevated TDN and ${\mathrm{NO}}_{3}^{-}$ losses from the fine, but not coarse, soil during plant‐microbe asynchronies. This could be related to the higher moisture content of the fine soil (~twice as high; Juice et al. 2022) which supported more N cycling microbial activity during the spring than in the drier coarse soil (Juice et al. 2022). Because the fine soils experienced increased duration of plant‐microbe asynchronies characterized by more frequent thawing during the period of plant dormancy (see H1 above), it is also possible that warming elevated fine soil N losses through the action of freeze–thaw cycles which can mobilize soil nutrients (Wipf et al. 2015). Effects of warming and snow exclusion on loss of Mg and Al during plant‐microbe asynchronies also differed by soil type, suggesting that the modification of climate impacts by soils may affect a wide range of biogeochemical processes.

*Asynchrony losses increased with asynchrony duration, particularly on coarse soils*.

In general, our hypothesis was supported by our data in which asynchrony losses of nutrients increased with duration of asynchronies (Figure 3: 2015 DOC, 2015 TDN, ${\mathrm{NO}}_{3}^{-}$, 2015 fine soil Mg), with greater losses from coarse soils (Figure 3: 2015 DOC, TDN, ${\mathrm{NO}}_{3}^{-}$, 2014 Ca, 2015 Mg). However, there was variation by year and soil type and some instances in which duration of asynchronies correlated with reduced nutrient leaching (Figure 3: 2014 coarse soil TDN, 2015 fine soil ${\mathrm{NO}}_{3}^{-}$, 2014 fine soil ${\mathrm{PO}}_{4}^{3-}$, 2015 coarse soil Mg). In some instances, the direction of coarse and fine soil leaching of a given nutrient opposed each other (Figure 3; 2014 TDN, 2015 ${\mathrm{NO}}_{3}^{-}$, 2014 ${\mathrm{PO}}_{4}^{3-}$, 2015 Mg) or switched direction across years (coarse soil TDN losses). Of the nutrients we analyzed, N losses were most consistently affected by plant‐microbe asynchronies, displaying significant relationships both years of the study as opposed to the other nutrients which were only affected one year, or in the cases of ${\mathrm{NH}}_{4}^{+}$ and Al not at all (Figure 3, Figure S5).

Our finding of increased nutrient losses positively correlating to the duration of plant‐microbe asynchronies aligns with prior evidence that the timing of plant and microbial phenology critically controls ecosystem nutrient loss (Brooks et al. 1998; Muller and Bormann 1976; Schmidt et al. 2007). The variations in nutrient losses by soil type that we observed also coincide with previous findings that soil characteristics alter snow melt chemistry (Fahey 1979) and may explain differential responses of N leaching to soil freezing (e.g., Groffman et al. 2011). In addition to augmented nutrient losses that can affect water quality and forest productivity, protracted vernal asynchronies can have lagged effects throughout the growing season, including reduced peak photosynthesis (Ouimette et al. 2018). Although the mechanisms driving such reductions in photosynthesis are unclear, the increased nutrient loss we observed under longer asynchronies in the present study provides a potential explanation.

## 5 Conclusion

To understand how soil characteristics interact with climate treatments to modify the synchrony of plant and microbial activity and associated soil nutrient losses, we conducted a multi‐year large forest sapling mesocosm experiment that imposed warming and snow exclusion treatments on two different soils. Our results address how changes to the winter climate in the northeast USA may impact overwinter and early spring nutrient losses from temperate forest ecosystems. We found that soil properties modified the effects of climate change on organismal phenology and ecosystem biogeochemistry such that the occurrence and magnitude of both plant‐microbe asynchronies and associated biogeochemical losses often depended on soil type. Specifically, we found that warming increased the duration of plant‐microbe asynchronies, but that the size of the effect varied by soil type, likely due to soil properties altering the nature of the freezing and thawing dynamics in the spring. Nutrient losses during the asynchrony period also varied across soil type, with warming and snow exclusion having a greater effect on leaching from fine soils. Importantly, we found that most of the annual soil nutrient losses we measured occurred during the asynchrony period, demonstrating its biogeochemical significance. Furthermore, longer asynchronies were often associated with greater nutrient losses, although with variation over time and by nutrient. Overall, our results show that soil characteristics contributed to variation in the duration of plant‐microbe asynchronies, as well as the incidence and extent of C and nutrient loss experienced as a function of the duration of the asynchrony. Our finding that warming increased asynchrony duration provides additional evidence that temporal shifts due to climate change may decouple plant and microbial activity. This decoupling led to increased loss of C, N, and other nutrients, which could impact plant productivity, ecosystem‐level C storage, and ultimately represent a terrestrial ecosystem feedback to the climate system.

## Author Contributions

**Stephanie M. Juice:** conceptualization, data curation, formal analysis, funding acquisition, investigation, methodology, visualization, writing – original draft, writing – review and editing. **Paul G. Schaberg:** conceptualization, investigation, methodology, project administration, writing – review and editing. **Alexandra M. Kosiba:** conceptualization, investigation, methodology, writing – review and editing. **Carl E. Waite:** conceptualization, investigation, methodology, project administration, writing – review and editing. **Gary J. Hawley:** conceptualization, investigation, methodology, project administration, writing – review and editing. **Deane Wang:** conceptualization, investigation, methodology, project administration, writing – review and editing. **Julia N. Perdrial:** investigation, methodology, resources, supervision, writing – review and editing. **E. Carol Adair:** conceptualization, formal analysis, funding acquisition, investigation, methodology, project administration, resources, supervision, writing – review and editing.

## Conflicts of Interest

The authors declare no conflicts of interest.

## Supporting information


**Data S1:** gcb70447‐sup‐0001‐supinfo.pdf.

## Data Availability

The data that support the findings of this study are openly available in Zenodo at https://doi.org/10.5281/zenodo.16783117.
