# Supplementary material for: Warming, Snow Exclusion, and Soil Type Alter the Timing of Plant and Soil Activity and Associated Nutrient Losses
Source: Glob Chang Biol. 2025 Aug 19;31(8):e70447. doi: 10.1111/gcb.70447 (PMC12365583; doi:10.1111/gcb.70447)
Supplement: Supplementary file 1 — Data S1: gcb70447‐sup‐0001‐supinfo.pdf. [file GCB-31-e70447-s001.pdf]

**Supporting Information.** Stephanie M. Juice, Paul G. Schaberg, Alexandra M. Kosiba, Carl E. Waite, Gary J. Hawley, Deane Wang, Julia N. Perdrial, and E. Carol Adair. Warming, snow exclusion, and soil type alter the timing of plant and soil activity and associated nutrient losses.

**Supplementary Tables**

**Table S1.** Physical and chemical properties of the soils used in the in-field forest sapling mesocosm experiment in South Burlington, VT. Data are means (standard errors) where available.

| Soil Property | Coarse | Fine |
|---|---|---|
| Bulk density (g $cm^{-3}$) | 1.724 | 1.498 |
| WHC (%) | 9.6 | 14.1 |
| CEC (meq 100 $g^{-1}$) | 11.08 | 0.98 |
| Clay (%) | 3.07 | 6.13 |
| Silt (%) | 10.23 | 6.50 |
| Sand (%) | 86.70 | 87.37 |
| Fine gravel >2 mm (%) | 44.97 | 17.80 |
| pH | 7.639 (0.173) | 6.156 (0.193) |
| Ca (mg $kg^{-1}$) | 1773.75 (68.74) | 70.00 (4.68) |
| P (mg $kg^{-1}$) | 4.50 (0.80) | 1.55 (0.22) |
| K (mg $kg^{-1}$) | 42.83 (4.36) | 48.79 (3.89) |
| Mg (mg $kg^{-1}$) | 35.36 (1.22) | 10.69 (0.90) |
| Na (mg $kg^{-1}$) | 5.83 (0.31) | 4.93 (0.33) |
| Al (mg $kg^{-1}$) | 7.74 (0.15) | 11.16 (0.44) |
| Fe (mg $kg^{-1}$) | 9.07 (0.57) | 4.27 (0.45) |
| Mn (mg $kg^{-1}$) | 20.26 (0.73) | 12.91 (0.94) |
| S (mg $kg^{-1}$) | 32.16 (1.31) | 6.91 (1.07) |
| % C | 0.698 (0.039) | 0.325 (0.033) |
| % N | 0.045 (0.004) | 0.031 (0.003) |
| C:N | 15.891 (0.851) | 10.520 (0.350) |

WHC: water holding capacity; CEC: cation exchange capacity; Ca: calcium, P: phosphorus; K: potassium; Mg: magnesium; Na: sodium, Al: aluminum; Fe: iron; Mn: manganese; S: sulfur; C: carbon; N: nitrogen.

Data are from samples collected in 2013 before initiation of climate treatments with the following exceptions: soil texture (% clay, silt, sand and fine gravel) was measured on soils collected in 2014, and pH and CEC were measured on soils collected in 2015.

**Table S2.** Rooting depth characteristics and relative location of the South Burlington, VT experimental site in relation to tree species ranges for saplings planted in the mesocosms.

| Location | Shallow rooted | Deep rooted |
|---|---|---|
| At south of range | paper birch<br>(*Betula papyrifera* Marshall) | quaking aspen<br>(*Populus tremuloides* Michx.) |
| At north of range | black cherry<br>(*Prunus serotina* Ehrh) | American chestnut<br>(*Castanea dentata* (Marshall) Borkh.) |

**Table S3.** Bud and leaf development descriptions for assessments of sapling spring phenology in the in-field forest sapling mesocosm experiment in South Burlington, VT (West and Wein 1971).

| Stage | Defining Characteristics |
|---|---|
| 0 | Buds dormant with scales closed |
| 1 | Buds display silver/green tip, greenness between scales |
| 2 | Buds green and tight or scales slightly separated |
| 3 | Buds expanding, leaves unfolding |
| 4 | Internodes/petioles visible, leaves hanging but not enlarged |
| 5 | Internodes/petioles visible, leaves enlarged |

**Supplementary Figures**

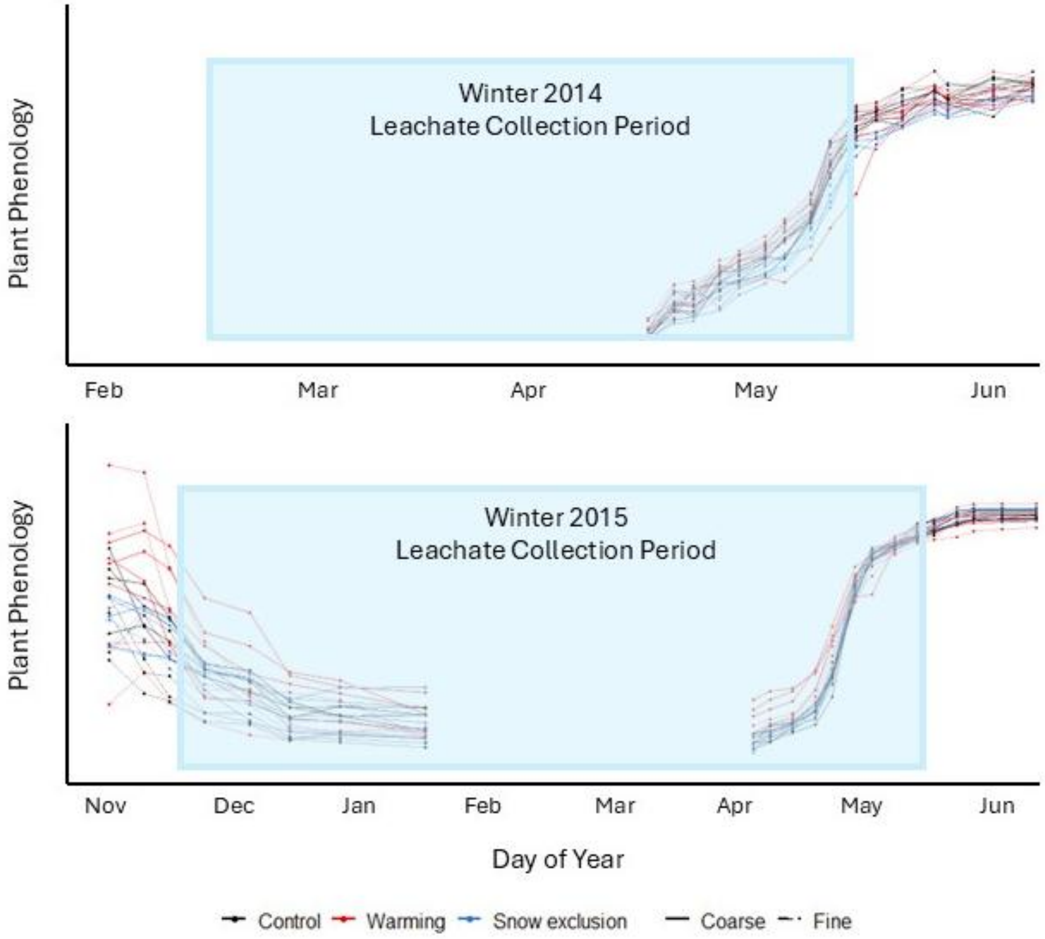


**Fig. S1** Conceptual diagram showing how the period of leachate collection bounded the determination of plant microbe asynchrony days for use in this study. Microbial activity during plant dormancy can occur at any point from the diminution of plant activity in autumn (plant downturn day) until leaf expansion in spring (plant stabilization date). To accurately assess the relationship between asynchrony duration and associated soil nutrient leaching, we had to constrain our analysis of the asynchrony duration to the period that aligned with the mesocosm leachate collection. As a result, our estimates of asynchrony duration and the associated biogeochemical losses are likely conservative.

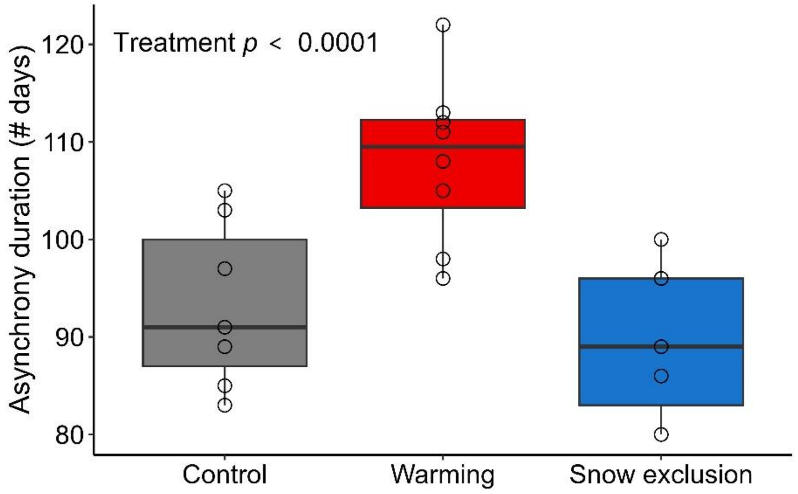


**Fig. S2.** Total asynchrony duration for each climate treatment measured from fall 2014 until spring 2015 in an in-field forest sapling mesocosm experiment in South Burlington, VT. For this analysis, we calculated asynchrony duration as the number of days with daytime soil temperatures ≥4 °C at 5 cm depth while plants were dormant (from the mesocosm average plant downturn day in the fall until the mesocosm average plant stabilization date in the spring). Open circles represent data points.

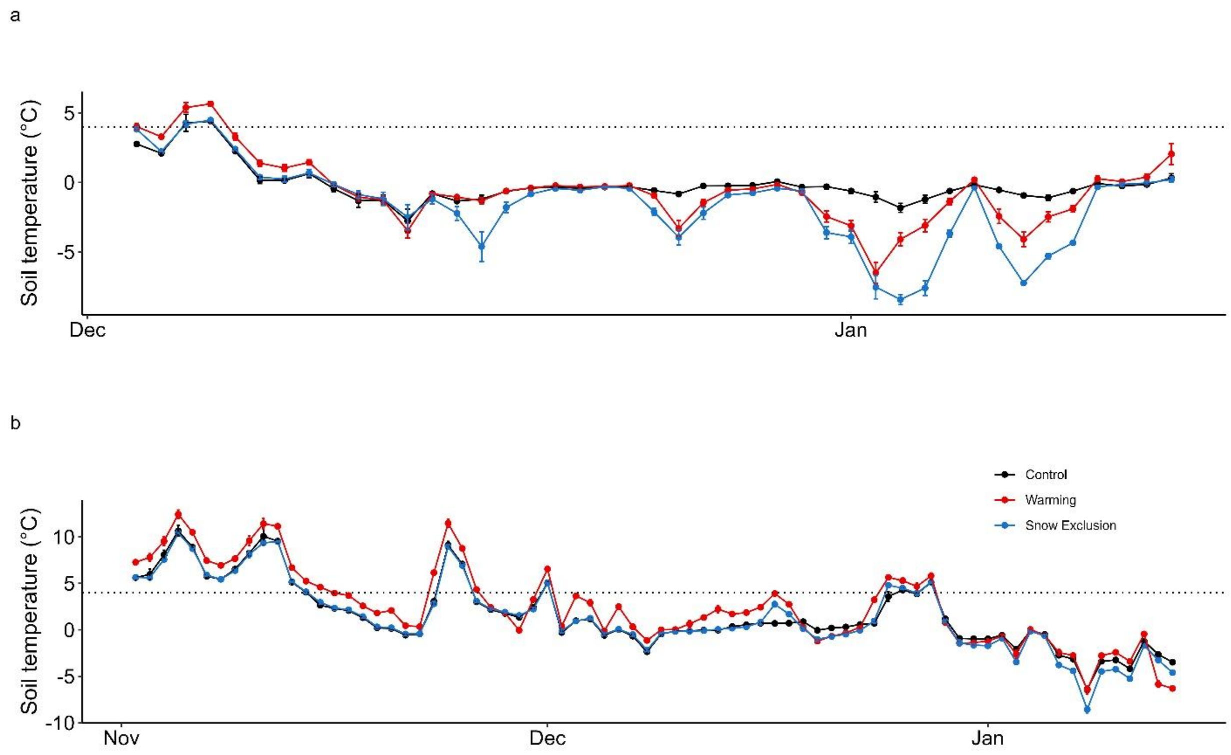


**Fig. S3** Daytime mean soil temperatures in fall (a) 2014 and (b) 2015 were measured at 5 cm depth. The dotted horizontal line in panels a and b marks 4 °C, the soil temperature at which rapid biological activity is thought to begin (Groffman et al. 2012). Note that the panels have different x and y axis limits.

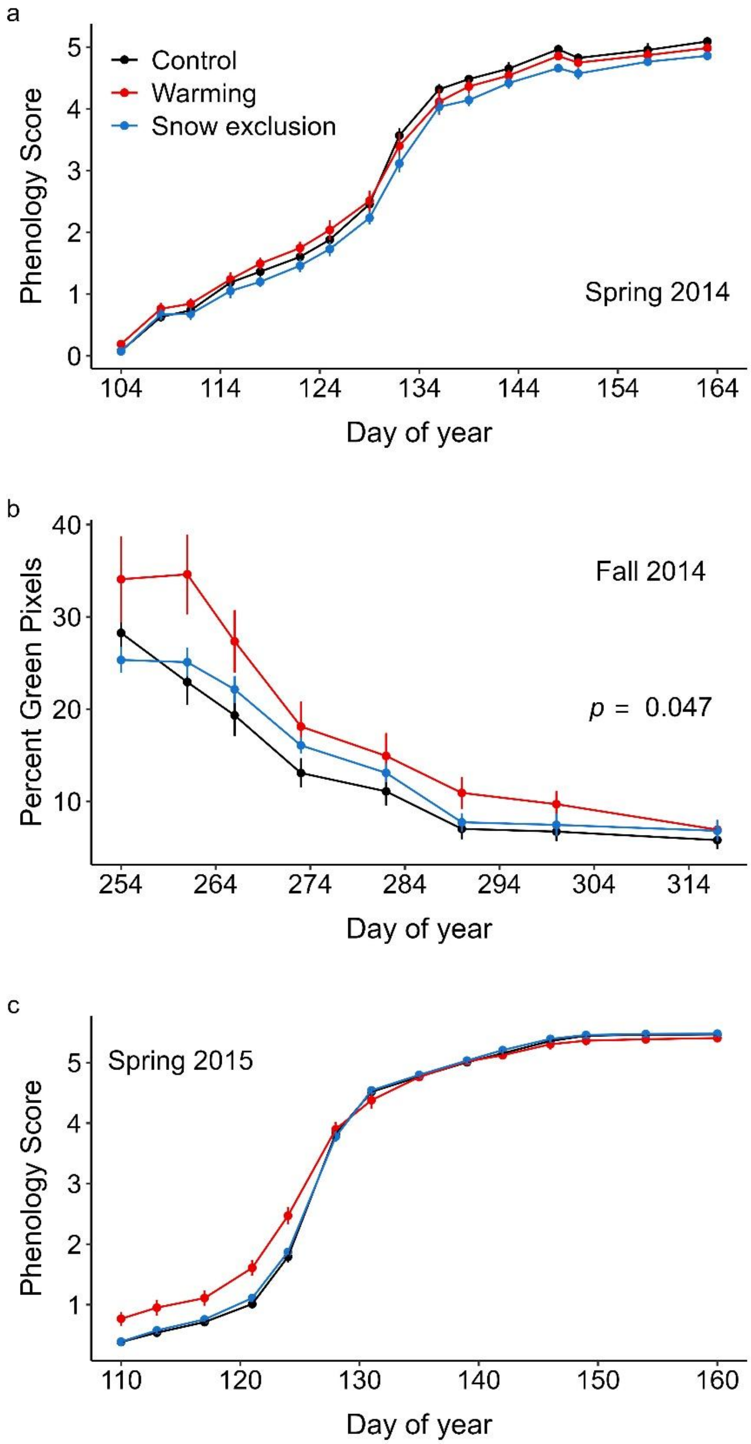


**Fig. S4** Average plant phenology by climate treatment in (a) spring 2014, (b) fall 2014, and (c) spring 2015. Spring phenology was scored on a scale of 0-5 as described in Table S3 (West and Wein 1971). Fall phenology was assessed by quantifying the percentage of green pixels in digital photographs. Phenology was significantly related to climate treatments only in fall 2014 (p-value shown on figure).

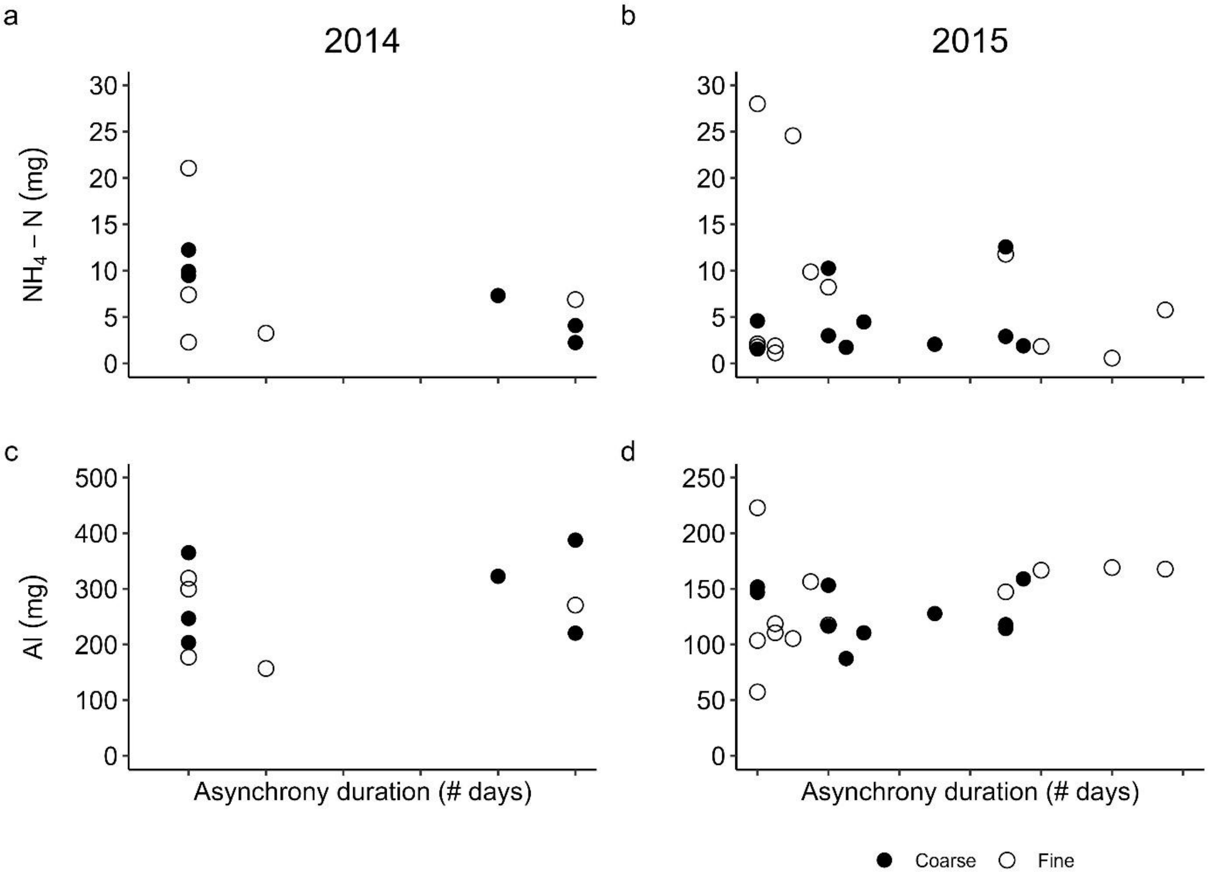


**Fig. S5** Relationship between asynchrony length and soil type with leachate water loss of $\mathbf{NH_4^+}$ **(a)** and **(b)** and Al **(c)** and **(d)** during plant-microbe asynchronies in an in-field forest sapling mesocosm experiment in South Burlington, VT in 2014 (left column) and 2015 (right column). We calculated asynchrony length as the number of days with daytime soil temperatures ≥4 °C at 5 cm depth while plants were dormant between mesocosm leachate collection dates. No significant regressions between analyte loss and asynchrony length were detected. See table 2 for associated analysis of deviance results.

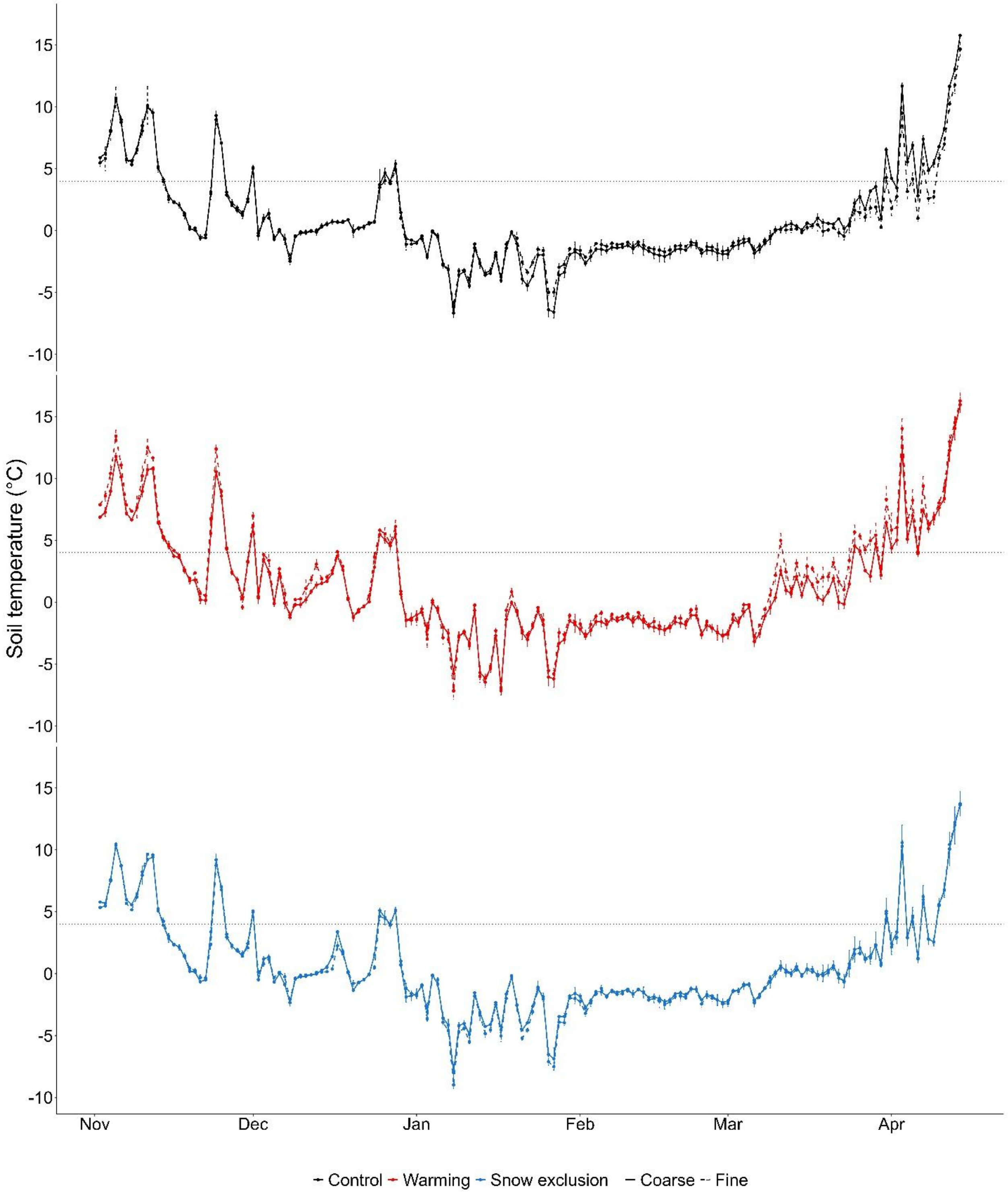


**Fig. S6** Daytime mean soil temperatures in winter 2015 (from November 2014 through April 2015) measured at 5 cm depth for each soil type and climate treatment. The dotted horizontal line in each panel marks 4 °C, the soil temperature at which rapid biological activity is thought to begin (Groffman et al. 2012).

## References

Groffman, P. M., Rustad, L. E., Templer, P. H., Campbell, J. L., Christenson, L. M., Lany, N. K., Socci, A. M., Vadeboncoeur, M. A., Schaberg, P. G., Wilson, G. F., Driscoll, C. T., Fahey, T. J., Fisk, M. C., Goodale, C. L., Green, M. B., Hamburg, S. P., Johnson, C. E., Mitchell, M. J., Morse, J. L., Pardo, L. H., & Rodenhouse, N. L. (2012). Long-term integrated studies show complex and surprising effects of climate change in the northern hardwood forest. *BioScience, 62*(12), 1056-1066. https://doi.org/10.1525/bio.2012.62.12.7

West, N. E., & Wein, R. W. (1971). A plant phenological index technique. *BioScience, 21*(4), 116-117.
